# Surgical resection of vascular-invasive late-stage hepatocellular carcinoma following transarterial chemoembolization combined with lenvatinib and tislelizumab: Two case reports and literature review

**DOI:** 10.1097/MD.0000000000042973

**Published:** 2025-06-20

**Authors:** Liying Qian, Kangze Wu, Gang Xiao, Zhihong Shen

**Affiliations:** a Department of Hepatobiliary Surgery, Shaoxing People’s Hospital, Shaoxing, People’s Republic of China; b The First Affiliated Hospital, Shaoxing University, Shaoxing, People’s Republic of China.

**Keywords:** gallbladder, HCC, hepatic vein, immunohistochemistry, immunotherapy, lenvatinib, liver metastasis, TACE

## Abstract

**Rationale:**

Hepatocellular carcinoma (HCC) with hepatic vein invasion poses significant treatment challenges and is associated with poor prognosis. Recent studies suggest that a combination of transarterial chemoembolization (TACE), targeted therapy, and immunotherapy may downstage advanced tumors, making surgical resection possible.

**Patient concerns:**

A 61-year-old female presented with a 3.7 × 3.1 cm hepatic mass invading the hepatic veins. A 75-year-old male exhibited a 6.7 × 5.1 cm liver tumor involving the right hepatic vein.

**Diagnoses:**

Both patients were diagnosed with advanced HCC with vascular invasion, confirmed by contrast-enhanced imaging and elevated alpha-fetoprotein (AFP) levels.

**Interventions:**

In case 1, the patient received 2 rounds of TACE and 3 cycles of lenvatinib plus tislelizumab. In case 2, the patient underwent 1 TACE session and 2 cycles of the same combination therapy. Both cases showed significant tumor shrinkage, allowing subsequent R0 surgical resection.

**Outcomes:**

Pathological evaluation following surgery revealed a major pathological response in both patients. Postoperative recovery was uneventful, and both patients remained disease-free during follow-up.

**Lessons:**

The combination of TACE, lenvatinib, and tislelizumab may offer an effective multimodal strategy for converting unresectable HCC with vascular invasion into resectable disease, potentially improving long-term outcomes.

## 1. Introduction

Liver cancer severely impacts human health, ranking as the sixth most common malignant tumor globally and the third leading cause of cancer-related deaths. Hepatocellular carcinoma (HCC) accounts for approximately 75% to 85% of liver cancer cases.^[1]^ The survival rates for HCC patients are low, with reported average 1-year survival rates below 50% and 5-year survival rates below 10%. This underscores the urgency of enhancing early detection and more effective treatments to improve the prognosis of HCC patients.^[2]^

HCC located in vascularly complex regions poses substantial surgical challenges due to its propensity for vascular invasion, which increases the risk of postoperative recurrence and metastasis. Such cases are often classified as stage C in the Barcelona Clinic Liver Cancer (BCLC) staging system, indicating advanced disease for which surgical treatment is generally not recommended. However, neoadjuvant therapy is often required prior to surgery to eradicate microscopic foci, reduce recurrence and metastasis rates, and extend survival periods.

Studies have demonstrated that transarterial chemoembolization (TACE) can create surgical opportunities for patients with initially unresectable liver cancer, translating into survival benefits.^[3]^ Additionally, the combination of systemic antitumor treatments, including hepatic arterial infusion chemotherapy,^[4]^ targeted therapies,^[5,6]^ and immunotherapy,^[7]^ can significantly increase the success rate of conversion therapy. Advanced techniques, such as 2-step liver resection strategies like associating liver partition and portal vein ligation for staged hepatectomy,^[8]^ have further improved outcomes. These approaches collectively enhance resectability, reduce postoperative risks, and offer promising avenues for improving prognosis in patients with intermediate to advanced HCC.

Herein, we report 2 cases of HCC located in vascularly complex regions. The first case involves a solitary segment I HCC situated between the inferior vena cava trunk, the right hepatic vein, and the middle hepatic vein. The second case features an HCC larger than 5 cm located between the middle and right hepatic veins, with poorly defined boundaries relative to the surrounding vasculature. Both cases were treated with TACE in combination with lenvatinib and tislelizumab for targeted immunotherapy. Following these treatments, the tumor volumes were reduced, potentially creating conditions favorable for surgical resection. These cases provide a potential approach for the treatment of intermediate to advanced HCC, highlighting the role of TACE combined with systemic therapy to achieve conversion therapy followed by surgical resection.

## 2. Case report

### 2.1. Case 1

**Figure 1.**
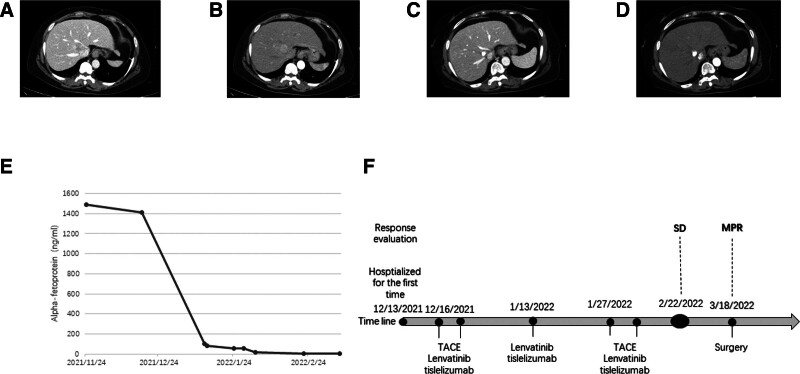
Changes in imaging and laboratory tests for case 1 during clinical treatment. Enhanced CT images before the introduction of TACE. In the portal venous phase (A) and on the unenhanced scan (B), a 3.7 × 3.1 cm mass is visible between the right hepatic vein and the middle hepatic vein. Enhanced CT images after the introduction of TACE. In the portal venous phase (C) and on the unenhanced scan (D), coagulative necrosis of the lesion is visible, with clearer hepatic vein contours than before. Changes in AFP levels during TACE combined with targeted immunotherapy (E). (F) Timeline of the treatment process. TACE = transarterial chemoembolization.

A 61-year-old female was admitted to the hospital due to a liver mass discovered during an abdominal ultrasound examination. The patient had a history of chronic hepatitis B virus infection for 3 years and had been taking antiviral medication long-term. Laboratory results at the time of admission are presented in Table 1. After admission, an abdominal enhanced computed tomography scan revealed a 3.7 × 3.1 cm mass located between the right hepatic vein and the middle hepatic vein (Fig. 1A, B). No metastasis was observed in other parts. The alpha-fetoprotein (AFP) level at admission was 1414 ng/mL. Liver function was classified as child–pugh grade A, and the BCLC staging was stage C. Following discussion, the patient underwent treatment with TACE, lenvatinib, and tislelizumab. The TACE procedure included the injection of oxaliplatin 100 mg, fluorouracil 500 mg, and epirubicin 30 mg.

**Table 1:** Admission laboratory parameters of the patient.

	Case 1	Case 2
Alanine aminotransferase	81.6	20.1
Aspartate aminotransferase	212.5	24.8
Total bilirubin	16.9	10.8
Direct bilirubin	3.8	2.5
Albumin	39.7	42.3
Prothrombin time	14.5	12.4
Alpha-fetoprotein	1414	2.84
White blood cell	13.24	5.31
Hemoglobin	122	145
Viral hepatitis type B	Positive	Positive
Abnormal prothrombin	None	7987.03

After receiving 2 rounds of TACE treatment and 3 cycles of tislelizumab therapy, the patient was reevaluated with an abdominal enhanced computed tomography scan. The scan suggested coagulative necrosis of the lesion, with clearer contours of the hepatic veins compared to before, indicating a shrinkage of the active tumor area (Fig. 1C, D). During the treatment period, the AFP level decreased to 4.74 ng/mL (Fig. 1E). According to the RECIST 1.1 criteria, the target lesion response was classified as stable disease (SD), and according to the mRECIST criteria, it was also classified as SD.

The patient subsequently underwent curative resection for liver cancer. Postoperative pathological examination found that the tumor was mainly coagulative necrotic, with some atypical cell nests around. The postoperative pathological assessment indicated major pathologic response (MPR), and the patient’s condition was stable postoperatively (Fig. 1F). During a 2-year follow-up period, no significant recurrence was observed.

### 2.2. Case 2

**Figure 2.**
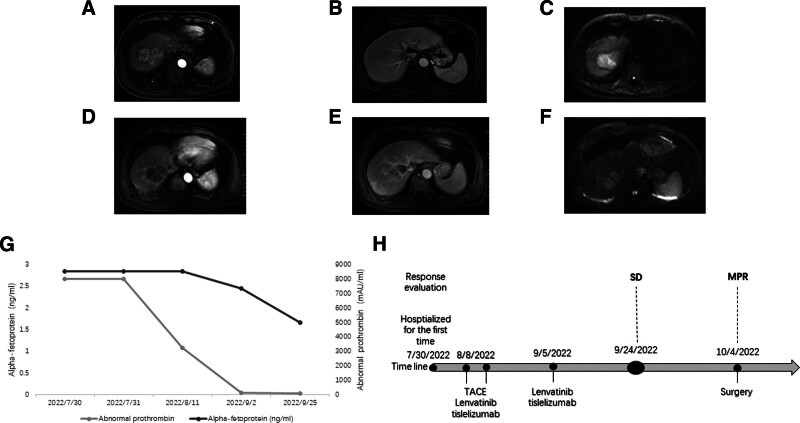
Changes in imaging and laboratory tests for case 2 during clinical treatment. Enhanced MRI images before the introduction of TACE. In the arterial phase (A), venous phase (B), and diffusion-weighted imaging (C), a 6.7 × 5.1 cm tumor is visible with unclear boundaries with the middle hepatic vein and the right hepatic vein, showing restricted diffusion. Enhanced MRI images after the introduction of TACE. In the arterial phase (D), venous phase (E), and diffusion-weighted imaging (F), the lesions appear smaller than before, with some areas still considered to be active, and the venous contours are relatively clear. Changes in AFP and abnormal prothrombin enzyme levels during TACE combined with targeted immunotherapy (G). (H) Timeline of the treatment process. TACE = transarterial chemoembolization.

A 75-year-old male was admitted to the hospital due to a large occupying lesion in the right liver with intrahepatic small nodules found during an abdominal ultrasound examination. The patient had a history of chronic hepatitis and had been treated with antiviral drugs. Laboratory results at the time of admission are presented in Table 1. After admission, the patient underwent an upper abdominal enhanced magnetic resonance imaging (MRI) scan, which revealed 2 tumors in the liver. The larger 1 measured 6.7 × 5.1 cm, with unclear boundaries with the middle hepatic vein and right hepatic vein, while the other tumor was approximately 1.2 cm and located in the posterior lobe of the right liver, with both showing diffusion restriction (Fig. 2A–C). No metastasis was observed in other parts. The AFP level at admission was 2.84 ng/mL, with an abnormal prothrombin enzyme at 7987.03 mAU/mL. Liver function was classified as child–pugh grade A, and the BCLC staging was stage C. After discussion, the patient received treatment with TACE, lenvatinib, and tislelizumab. The TACE procedure included the injection of oxaliplatin 100 mg, fluorouracil 500 mg, and epirubicin 30 mg.

After receiving 1 round of TACE treatment and 2 cycles of tislelizumab therapy, the patient was reassessed with an upper abdominal enhanced MRI scan. The results showed 2 tumors in the liver, with diffusion limitation. The larger tumor had reduced to approximately 6.2 × 4.3 cm, suggesting partial active lesions, and the venous contours were relatively clear (Fig. 2D–F). During the treatment period, the patient’s AFP level stabilized at 1.66 ng/mL, and the abnormal prothrombin enzyme decreased to 87.78 mAU/mL (Fig. 2G). According to the RECIST 1.1 criteria, the target lesion response was classified as SD, and according to the mRECIST criteria, it was also classified as SD.

The patient subsequently underwent curative resection for liver cancer. Postoperative pathological examination revealed the larger tumor as moderately to poorly differentiated hepatocellular carcinoma with extensive necrosis, while the smaller tumor was considered completely necrotic. The postoperative pathological assessment indicated a MPR, and the patient’s condition was stable postoperatively (Fig. 2H).

## 3. Discussion

We reported 2 rare cases of HCC involving the middle hepatic vein and the right hepatic vein that were successfully treated with TACE combined with lenvatinib and tislelizumab as conversion therapy. Postoperative pathological assessments in both cases revealed a MPR, highlighting the effectiveness of this multimodal approach.

TACE is widely recognized as a standard treatment for intermediate and advanced liver cancer. Approximately 8% to 18% of patients treated with TACE achieve tumor conversion to a surgically resectable state. For patients with early-stage disease managed with TACE, the 5-year survival rate ranges from 24.9% to 57%, with some achieving even higher survival rates.^[9]^ These outcomes emphasize TACE’s role in providing long-term clinical benefits and surgical opportunities for patients initially deemed unsuitable for curative surgery.

Lenvatinib, a multikinase inhibitor targeting VEGF receptors 1 to 3, FGF receptors 1 to 4, PDGF receptor α, RET, and KIT, offers an objective response rate (ORR) of 18.8%, significantly surpassing the traditional treatment with sorafenib.^[10]^ Recent strategies in conversion therapy frequently combine targeted therapy with immunotherapy. For instance, among 60 patients with unresectable cancer treated with tyrosine kinase inhibitors (TKIs) and immune checkpoint inhibitors (ICIs), 11 patients experienced tumor conversion to a resectable state.^[11]^ Another study demonstrated that 42.4% of HCC patients with portal vein tumor thrombosis achieved surgical resectability following TKI–ICI combination therapy. These findings underscore the feasibility and promise of combination therapy in conversion treatment.^[12]^

In recent years, TACE combined with lenvatinib and ICIs has emerged as a promising strategy, achieving superior therapeutic outcomes and becoming a focal point in HCC conversion therapy. Research by Cai et al demonstrated that patients receiving TACE, lenvatinib, and ICIs had significantly longer overall survival (median 16.9 months vs 12.1 months, *P* = .009) and progression-free survival (median 7.3 months vs 4.0 months, *P* = .002) compared to those receiving only TACE and lenvatinib. The combination therapy also resulted in higher ORR (56.1% vs 32.5%, *P* = .033) and disease control rate (85.4% vs 62.5%, *P* = .019).^[13]^

Additional studies have evaluated the efficacy of TACE combined with lenvatinib and PD-1 inhibitors in patients with unresectable HCC. Reported outcomes include progression-free survival of 11.4 to 13.3 months and overall survival of 23.6 to 24.0 months.^[14,15]^ A phase II clinical trial further highlighted the potential of this combination, with tumor reduction observed in 96.7% of patients and an ORR of 60.0%, surpassing results achieved with lenvatinib alone or lenvatinib combined with ICIs.^[16]^ The enhanced efficacy of this combination therapy is likely attributed to the ischemic necrosis induced by TACE, which generates novel antigens and enhances the tumor’s immunogenicity. Simultaneously, targeted immunotherapy remodels the tumor microenvironment, fostering a synergistic effect that amplifies the overall therapeutic response.

As a case report, this article illustrates the efficacy of TACE combined with targeted immunotherapy in an HCC patient with a tumor located at a specific anatomical site. Due to the limited sample size, comparisons with outcomes from alternative treatment modalities could not be performed. Future studies are warranted to build upon these findings and further investigate the therapeutic potential of this combination approach.

## 4. Conclusions

In summary, TACE combined with lenvatinib and tislelizumab represents a highly effective strategy for converting advanced HCC with vascular invasion into a surgically resectable state, offering the potential for R0 resection. Given the limited data available on this approach for HCC involving hepatic veins, these findings provide a strong foundation for further research and clinical application of this promising therapeutic combination.

## Author contributions

**Investigation:** Liying Qian, Kangze Wu, Gang Xiao.

**Methodology:** Liying Qian, Kangze Wu.

**Writing – original draft:** Liying Qian, Kangze Wu.

**Writing – review & editing:** Zhihong Shen.
